# Complete Genome Sequence of *Mycoplasma gallinaceum*

**DOI:** 10.1128/genomeA.00712-15

**Published:** 2015-07-02

**Authors:** Celia Abolnik, Amanda Beylefeld

**Affiliations:** Poultry Section, Department of Production Animal Studies, Faculty of Veterinary Science, University of Pretoria, Onderstepoort, South Africa

## Abstract

*Mycoplasma gallinaceum* strain B2096 8B was isolated from domestic chickens in South Africa. The 845,307-bp full genome was sequenced, assembled, and annotated.

## GENOME ANNOUNCEMENT

*Mycoplasma*, a genus within the class *Mollicutes*, is composed of the smallest known self-replicating prokaryotes. As a consequence of the evolutionary reduction in genome and cell size, mycoplasmas are deficient in genes controlling biosynthetic pathways and are reliant on their host for the provision of many essential nutrients (1).

*Mycoplasma gallinaceum* was first described in 1982 as a serovar that ferments glucose but not tetrazolium chloride, does not hydrolyze arginine or urea, and has a low G+C content of 29% (2). *M. gallinaceum* has not been considered as one of the major pathogenic avian mycoplasma species and has mostly been isolated from the upper respiratory tract of affected chickens, commonly colonizing the eyes and sinuses, but has also been found in the reproductive tract (3, 4). *Mycoplasma* strain B2096 8B was isolated in September 2014 in the Gauteng Province of South Africa from 62-week-old laying hens with typical mycoplasmal infection symptoms. Similar isolates have been made from chickens since 2005.

DNA extraction from the organism cultured in mycoplasma broth was performed as previously described (5). The genomic DNA library was sequenced using Illumina MiSeq technology for 600 cycles, generating approximately 1 Gb of data (Inqaba Biotech [Pty] Ltd., Pretoria, South Africa). The genome was assembled *de novo* from 6,229,108 reads, with an average length of 133.03 nucleotides (nt) and approximately 700× coverage in the CLC Genomics Workbench version 7.5.2. Contigs were aligned in the CLC Genome Finishing Tool version 1.4, visually inspected, and systematically joined after each realignment. The assembled genome was annotated in the NCBI Prokaryotic Genome Annotation Pipeline, and the functions of open reading frames (ORFs) were predicted on the BASys Web server (6). A maximum likelihood phylogenetic tree was constructed from the 16S rRNA sequences of B2096 8B and other avian mycoplasmas retrieved from GenBank using MEGA5 (7). Strain B2096 8B was subsequently identified as *M. gallinaceum*, with 99.38% nucleotide sequence identity to the 16S rRNA sequence of reference strain L24104 (data not shown).

The assembled circular genome of *M. gallinaceum* strain B2096 8B was 845,307 bp in length, with a G+C content of 28.38% and a genome size of 5.1 × 10^8^ Da. Proteins were identified with functions in energy production and conversion (1.6%), cell division and chromosome partitioning (0.5%), amino acid transport and metabolism (3%), nucleotide transport and metabolism (1%), carbohydrate transport and metabolism (4.3%), coenzyme metabolism (1.2%), lipid metabolism (0.3%), translation, ribosomal structure, and biogenesis (7.7%), transcription (1.4%), DNA replication, recombination, and repair (4.2%), cell envelope biogenesis/outer membrane (0.1%), posttranslational modification, protein turnover and chaperones (1.3%), inorganic ion transport and metabolism (0.9%), and proteins with a general function prediction only (4.6%). The functions of 65% of ORFs identified in *M. gallinaceum* are unknown. The complete genome of *M. gallinaceum* will assist in future studies aimed at unravelling the complex etiology of this pathogen in multifactorial disease in chickens.

### Nucleotide sequence accession number.

The complete sequence of *M. gallinaceum* strain B2096 8B was deposited in GenBank under the accession no. CP011021.
